# Inhibition of *P. aeruginosa* c-di-GMP phosphodiesterase RocR and swarming motility by a benzoisothiazolinone derivative†
†Electronic supplementary information (ESI) available: All experimental, spectroscopic details and supplemental figures. See DOI: 10.1039/c6sc02103d


**DOI:** 10.1039/c6sc02103d

**Published:** 2016-06-16

**Authors:** Yue Zheng, Genichiro Tsuji, Clement Opoku-Temeng, Herman O. Sintim

**Affiliations:** a Department of Chemistry , Purdue University , 560 Oval Drive , West Lafayette , IN 47907 , USA . Email: hsintim@purdue.edu; b Center for Drug Discovery , Purdue University , 720 Clinic Drive , West Lafayette , IN 47907 , USA; c Graduate Program in Biochemistry , University of Maryland , College Park , MD 20742 , USA

## Abstract

Various important cellular processes in bacteria are controlled by c-di-GMP, such as motility, biofilm formation and virulence factors production. C-di-GMP is synthesized from two molecules of GTP by diguanylate cyclases (DGCs) and its actions are terminated by EAL or HD-GYP domain phosphodiesterases (PDEs), which hydrolyze c-di-GMP to linear pGpG or GMP. Thus far the majority of efforts have been dedicated to the development of inhibitors of DGCs but not PDEs. This is probably because the old view was that inhibiting any c-di-GMP PDE would lead to biofilm formation, an undesirable phenotype. Recent data however suggest that some PDEs only change the localized (not global) concentration of c-di-GMP to increase bacterial virulence and do not affect biofilm formation. A challenge therefore is to be able to develop selective PDE inhibitors that inhibit virulence-associated PDEs but not inhibit PDEs that regulate bacterial biofilm formation. Using high throughput docking experiments to screen a library of 250 000 commercially available compounds against *E. coli* YahA (also called PdeL), a benzoisothiazolinone derivative was found to bind to the c-di-GMP binding site of YahA with favorable energetics. Paradoxically the *in silico* identified inhibitor (a benzoisothiazolinone derivative) did not inhibit the hydrolysis of c-di-GMP by YahA, the model PDE that was used in the docking, but instead inhibited RocR, which is a PDE from the opportunistic pathogen *P. aeruginosa* (PA). RocR promotes bacterial virulence but not biofilm dispersal, making it an ideal PDE to target for anti-virulence purposes. This newly identified RocR ligand displayed some selectivity and did not inhibit other *P. aeruginosa* PDEs, such as DipA, PvrR and PA4108. DipA, PvrR and PA4108 are key enzymes that reduce global c-di-GMP concentration and promote biofilm dispersal; therefore the identification of an inhibitor of a PA PDE, such as RocR, that does not inhibit major PDEs that modulate global c-di-GMP is an important step towards the development of selective c-di-GMP PDEs that could have interesting biomedical applications. The identified RocR ligand could also inhibit *P. aeruginosa* (PAO1) swarming but not swimming or biofilm formation. Rhamnolipid production was decreased, explaining the inhibition of swarming.

## Abstract 2

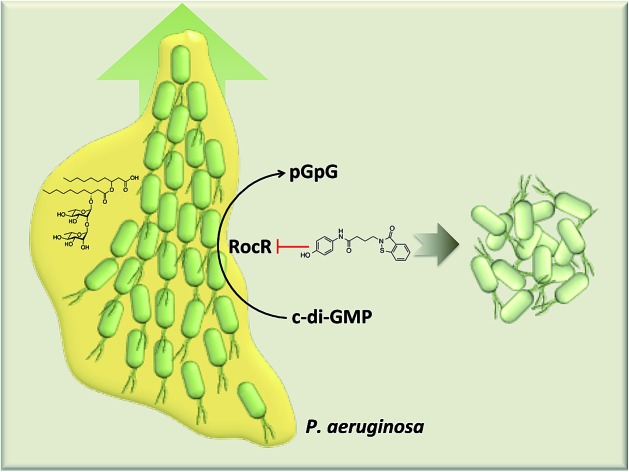
 *Pseudomonas aeruginosa* swarming motility can be inhibited with a small molecule inhibitor of c-di-GMP phosphodiesterase, RocR.

## Introduction

**Fig. 1:**
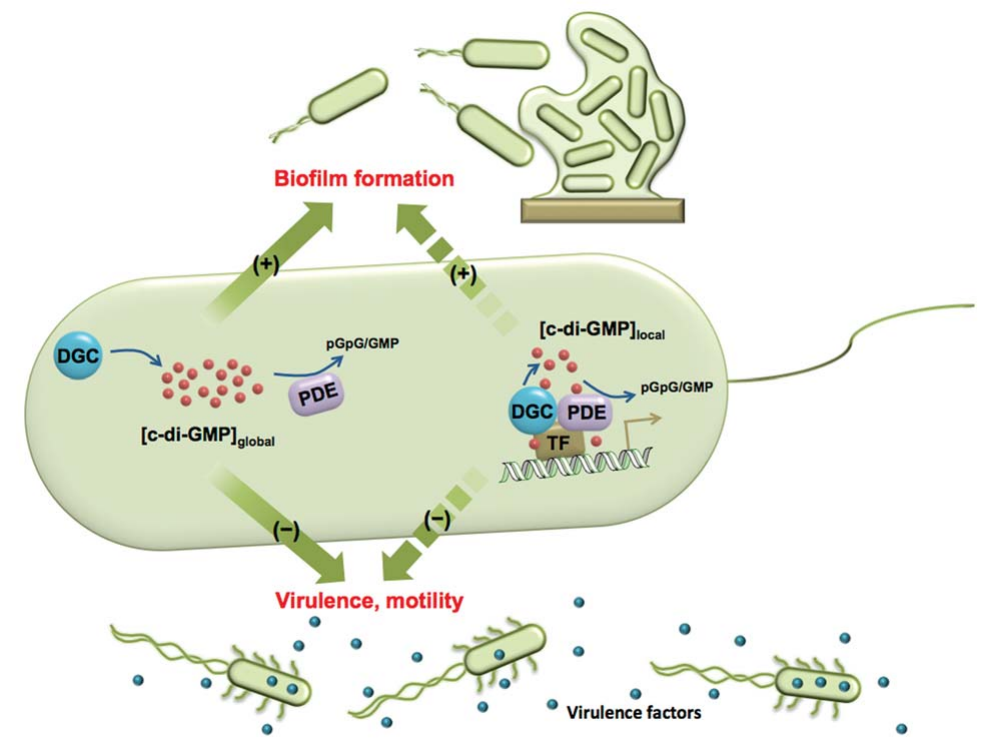
The global and local regulation of c-di-GMP signalling. Some DGCs and PDEs control the global concentration of c-di-GMP and regulate biofilm formation, motility and virulence factor production. Some DGCs and PDEs regulate c-di-GMP concentration in a localized pool and also function *via* direct interaction with effectors, such as transcription factors (TF). Although these DGCs and PDEs do not change global c-di-GMP concentration, they have a significant impact on bacterial phenotypes.

Cyclic dinucleotides are now acknowledged as important second messengers in bacteria.^1^ These second messengers also elicit an innate immune response in mammalian cells.^1^ The first cyclic dinucleotide bis-(3′-5′)-cyclic dimeric GMP (c-di-GMP) was discovered in *Gluconoacetobacter xylinum* by Benziman in 1987.^2^ At that time c-di-GMP was recognized as a regulator of cellulose synthesis. Following Benziman's seminal discovery, the field of cyclic dinucleotides went into a hiatus, only to be resurrected in the last decade, where the key roles played by c-di-GMP in signal transduction systems have been uncovered.^3^–^6^ In the majority of bacteria studied so far, the intracellular concentrations of c-di-GMP determine whether a bacterium chooses the mobile planktonic or the sedentary biofilm lifestyles (Fig. 1). At high intracellular concentrations, c-di-GMP promotes the production of exopolysaccharides and other adhesion factors to facilitate biofilm formation.^7^ On the other hand, c-di-GMP retards the expression of flagella and impedes bacterial swimming and swarming activities.^8^ C-di-GMP also represses the expression of the acute virulence genes.^9^ The intracellular concentration of c-di-GMP is controlled by its metabolic enzymes: diguanylate cyclase (DGC) and phosphodiesterase (PDE). DGCs dimerize two GTP into pppGpG, which is subsequently cyclized into c-di-GMP.^10^ PDEs hydrolyze c-di-GMP to either linear pGpG or two molecules of GMP, depending on the key residues in their active sites.^11^,^12^ The major product of EAL domain phosphodiesterase is pGpG and these enzymes only slowly hydrolyze pGpG to GMP.^13^ HD-GYP domain phosphodiesterase hydrolyzes c-di-GMP directly to GMP efficiently.^14^ Some c-di-GMP metabolism enzymes (both DGC and PDE) also contain sensory domains that sense various signals, such as oxygen,^15^,^16^ light,^17^,^18^ NO^19^,^20^ *etc.* to modulate enzymatic activity. Given that c-di-GMP binds to a plethora of downstream protein receptors^21^ and RNA riboswitches^22^ and regulates important bacterial behaviours, the roles of its metabolic enzymes are clearly worthy of attention.

The traditional view about c-di-GMP signaling has been that c-di-GMP synthases (GGDEF/GGEEF-domain proteins) increase intracellular concentration of c-di-GMP and increase biofilm formation whereas PDEs (EAL/HD-GYP domain proteins) decrease c-di-GMP concentration resulting in decreased biofilm formation and virulence. It has therefore been assumed that inhibiting c-di-GMP PDE would inadvertently promote biofilm formation, an undesirable phenotype. However, this is an oversimplification and it is emerging that there are nuances to c-di-GMP system in that gross intracellular concentration of the dinucleotide alone is not the sole determinant of a phenotype but rather the micro-concentrations of c-di-GMP and the relative localizations of c-di-GMP regulatory enzymes or adaptor proteins and/or binding RNAs dictate the phenotypic outcome.^23^–^25^ The nuances also arise from the fact that multitudes of DGCs and PDEs exist in a given bacteria. For example in *Pseudomonas aeruginosa*, there are 33 GGDEF domain proteins, 21 EAL domain proteins and 3 HD-GYP domain proteins (see Table S1† for the roles of some PDEs in *P. aeruginosa*).^26^ This begs the question why 33 different c-di-GMP synthases and 24 phosphodiesterases are needed for making or hydrolyzing one metabolite.^26^ A beautiful work by Lory and co-workers demonstrated that not all DGCs or GGDEF-domain proteins increase global intracellular c-di-GMP concentration and not all PDEs decrease global intracellular c-di-GMP concentration.^9^ For example in *P. aeruginosa*, the GGDEF-domain proteins PA0169 (SiaD), PA0285, PA0575 and PA2870 were shown to not affect biofilm formation even though some of these proteins, such as PA2870, did in fact have *in vitro* DGC activity.^9^ On the other hand, other GGDEF-domain proteins including PA0847, PA1107 (RoeA), PA1120 and PA3702 (WspR) possessed *in vitro* DGC activity and also affected biofilm formation.^9^ It therefore appears that the global regulation of c-di-GMP by DGCs occurs in a hierarchy and/or complex network. Similar observations were made about PDEs. Overexpression of *P. aeruginosa* PDEs such as PA2133, PA2200 and PA3825 resulted in decreased biofilm formation, as traditionally expected.^9^ However, some PDEs such as PA3947 (RocR) had no effect on biofilm formation despite its high *in vitro* cleavage activity.^9^ A slight increase in biofilm was observed with *pvrR* overexpression^9^ but others have also shown a decrease in biofilm formation,^27^ implying this could be context dependent.

Both the *rocR* (PA3947) mutant and overexpression strains did not affect *P. aeruginosa* biofilm formation, compared to wildtype.^9^ Since global c-di-GMP concentration affects biofilm formation, it can be inferred that RocR did not reduce global c-di-GMP concentration. Intriguingly, RocR was shown to possess one of the highest *in vitro* PDE activities.^9^,^28^ These contradictory observations could be rationalized in a situation whereby the PDE activity of RocR leads to a local but not global signaling. *In vivo* data indicates that *rocR* mutant of *P. aeruginosa* were avirulent in a mice model.^9^ Therefore RocR could be one of the PDEs that could be targeted with a small molecule without affecting global c-di-GMP concentration and/or biofilm formation.

C-di-GMP signaling network is very complicated and various effectors respond to the same diffusible molecule. The input of each DGC and PDE is different. How does the signal pass to downstream receptors correctly without crosstalk? A study by O'Toole's group suggested that physical interactions help inner membrane DGC GcbC to differentiate the effector protein LapD, which is a biofilm regulator.^29^ This localized interaction ensures fast communication, which is not disturbed by the rest of c-di-GMP signaling network.^29^ Another seminal study by Hengge and co-workers provided some insights into why some c-di-GMP metabolism enzymes do not change the intracellular concentrations of c-di-GMP, yet affect bacterial phenotype (a case of global *versus* localized signaling). In this study, the authors showed that in *Salmonella csgD* expression is not only regulated by total c-di-GMP concentration, but also the local DGC-PDE interactions; EAL domain YciR (PdeR) could act as trigger enzyme that inhibits *csgD* expression *via* direct contact with YdaM (DgcM) and MlrA (a transcription factor).^25^ Thus it is now clear that the cellular functions of c-di-GMP PDEs are not only achieved by decreasing global c-di-GMP concentration. In addition to the biomedical relevance of being able to specifically inhibit a virulence-associated c-di-GMP PDE, such as RocR (without increasing global c-di-GMP concentration), the ability to specifically inhibit each of these PDE enzymes in a specific bacteria, without affecting others of similar function, could help identify the cellular processes that are directly or indirectly regulated by these enzymes.

Thus far, there have been limited efforts to develop PDE inhibitors and the only reported inhibitors of PDEs are nucleotide/nucleoside-based, which are obviously poor probes due to cell permeation issues.^30^–^33^ Due to the dearth of PDE inhibitors we initiated a program to discover selective PDE inhibitors. We successfully identified a benzoisothiazolinone derivative that specifically inhibited RocR, but not some other PDEs. The compound also decreased the swarming motility of *P. aeruginosa*, in a dose-dependent manner, whereas biofilm formation was not increased.

## Results and discussion

### Identification of a RocR inhibitor

**Fig. 2:**
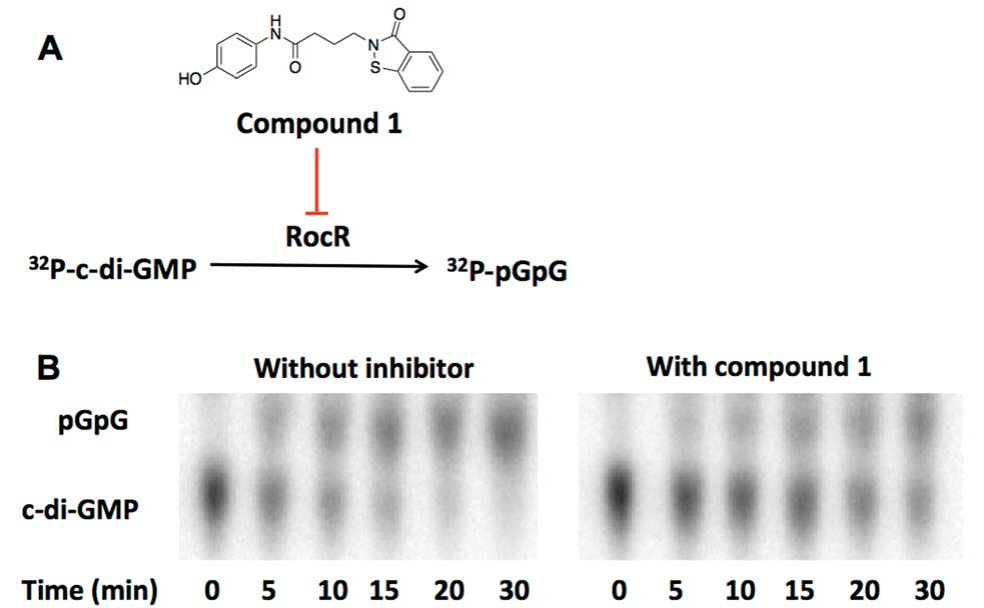
(A) The structure of compound **1**. Compound **1** inhibits the cleavage of ^32^P-c-di-GMP to ^32^P-pGpG by RocR. (B) The image of TLC plates of RocR cleavage with or without compound **1**. 50 μM c-di-GMP and 16 nM ^32^P-c-di-GMP were cleaved by 600 nM RocR in the presence or absence of 100 μM compound **1** at 37 °C for 30 min. Compound **1** significantly slowed down RocR cleavage.

We utilized high throughput docking to identify potential inhibitors of cyclic dinucleotide metabolic enzymes. Our ultimate goal was to find inhibitors against RocR, a *P. aeruginosa* PDE that has been shown to be important for virulence but the crystal structure of RocR reported to date did not have a bound ligand^34^ so this presented a challenge. On the other hand the crystal structure of YahA (also named PdeL), an EAL domain phosphodiesterase from *Escherichia coli*,^13^ in complex with its substrate, c-di-GMP, has been solved (PDB ; 4LJ3)^35^ so we decided to use this PDE for our docking experiment. Using the structures of 250 000 commercially available compounds, we performed the docking experiment against YahA and identified a handful of putative PDE binders. We then tested the ligands, which were identified as binders of PDE *in silico*, for inhibition of c-di-GMP cleavage by RocR^36^ and YahA. Only one compound (a benzoisothiazolinone derivative, compound **1**) could inhibit the cleavage of c-di-GMP by RocR (Fig. 2). Paradoxically this compound did not inhibit the cleavage of c-di-GMP by YahA from *E. coli* (Fig. S1†), although YahA was the protein that was used for the docking experiment. Nonetheless we were still excited about the ability of the “hit” compound to inhibit RocR since *P. aeruginosa* is an important human pathogen, which causes respiratory tract, urinary tract, wound and burns infections.^37^*P. aeruginosa* also colonizes medical devices and causes hospital-acquired infections. Cystic fibrosis (CF) patients are especially susceptible to this opportunistic pathogen. Thus small molecules that perturb any signaling system in *P. aeruginosa* could be useful for illuminating *P. aeruginosa* biology and in some cases could even have therapeutic value. C-di-GMP regulates some phenotypes of *P. aeruginosa* and RocR has been shown to be a major c-di-GMP PDE in this pathogen.^36^ As already stated, mutation of the *rocR* gene abolished the virulence of *P. aeruginosa* in mouse infection model.^9^

**Fig. 3:**
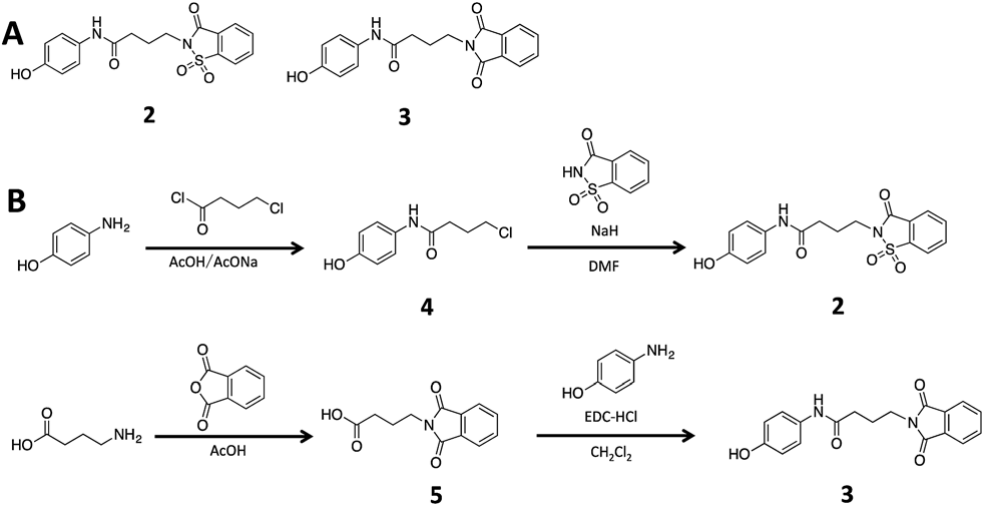
(A) The structures of compound **2** and **3**. (B) Synthesis scheme of compound **2** and **3**. See “Methods” for more details of synthesis.†

**Fig. 4:**
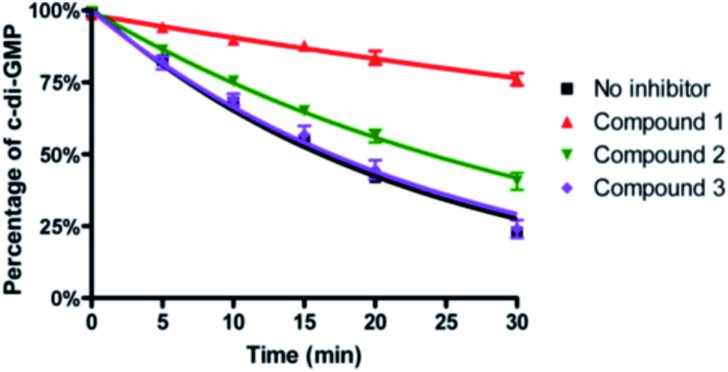
Inhibition of RocR reaction by compound **1**, **2** and **3**. 50 μM c-di-GMP and 16 nM ^32^P-c-di-GMP were cleaved by 400 nM RocR in the presence or absence of 100 μM small molecules at 37 °C for 30 min. Compared with compound **1**, compound **2** has a much weaker inhibition and compound **3** completely lost RocR inhibition activity.

To ascertain if the benzoisothiazolinone core was important for the inhibition of RocR, we synthesized two structurally similar compounds **2** and **3** (Fig. 3) and tested them for RocR inhibition. Compound **2** was not as potent as compound **1**, whereas **3** was not active (see Fig. 4), confirming the essentiality of the benzoisothiazolinone moiety for RocR inhibition. The benzoisothiazolinone unit is found in several biologically active molecules,^38^–^44^ including orally active drug leads against metabotropic glutamate subtype 2 receptor^33^ and phosphomannose isomerase;^34^ the PDE RocR adds to the growing list of enzymes that this pharmacophore inhibits.

### Selectivity of compound **1** inhibition

**Fig. 5:**
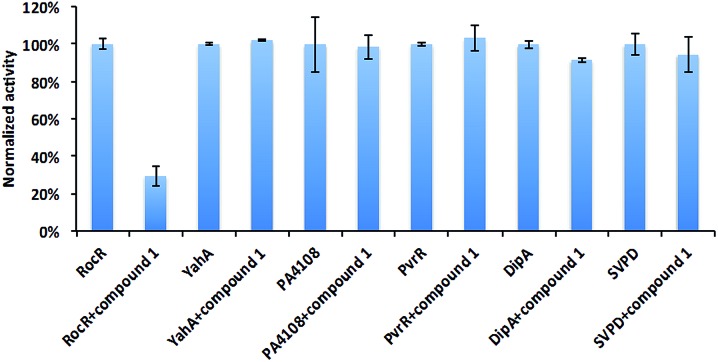
Effects of compound **1** on other PDEs. The enzymatic activities of different PDEs with or without compound **1** were analyzed by HPLC or bis-pNPP cleavage assays. Compound **1** did not significantly inhibit YahA from *E. coli*, PA4108, PvrR or DipA from *P. aeruginosa* and snake venom phosphodiesterase (SVPD) from *Crotalus atrox*. Experimental condition is described in Methods.†

Different PDEs make different contributions to the intracellular concentration of c-di-GMP either globally or locally. Mutating some genes that encode PDEs, such as DipA increases c-di-GMP concentration, and the biofilm of Δ*dipA* strain can not be easily dispersed.^11^ Thus an indiscriminate targeting of all c-di-GMP PDEs by compound **1** would lead to enhanced biofilm formation, which is undesirable. To exam the selectivity of compound **1**, we tested it against some other c-di-GMP PDEs (Fig. 5). As previously stated, it did not inhibit YahA (the enzyme used for initial docking) from *E. coli*. Compound **1** did not inhibit snake venom phosphodiesterase (SVPD) from *Crotalus atrox*, a promiscuous PDE that has been shown to cleave c-di-GMP.^45^ DipA and PvrR are EAL-domain c-di-GMP PDEs in *P. aeruginosa*. DipA is essential for biofilm dispersal^11^ and *pvrR* overexpression impedes biofilm formation.^27^ Compound **1** did not inhibit DipA and PvrR, as well as HD-GYP domain PDE PA4108. PA4108 mutation dramatically increased c-di-GMP level, reduced swarming motility and changed biofilm architecture.^46^ The discrimination displayed by compound **1**, especially not affecting the enzymatic activities of key PDEs that increase global c-di-GMP concentration is encouraging.

### Thermodynamic and kinetic parameters

**Fig. 6:**
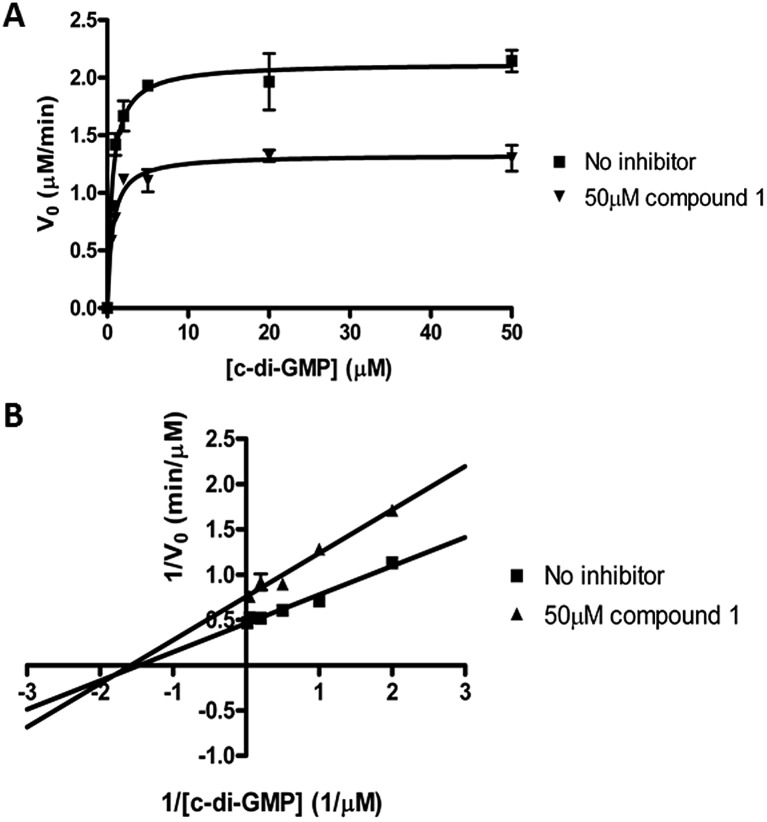
Kinetics of inhibition of RocR by compound **1**. (A) Michaelis–Menten kinetics. (B) Lineweaver–Burk plot showed that compound **1** is a non-competitive inhibitor. The experimental conditions are described in ESI.†

**Fig. 7:**
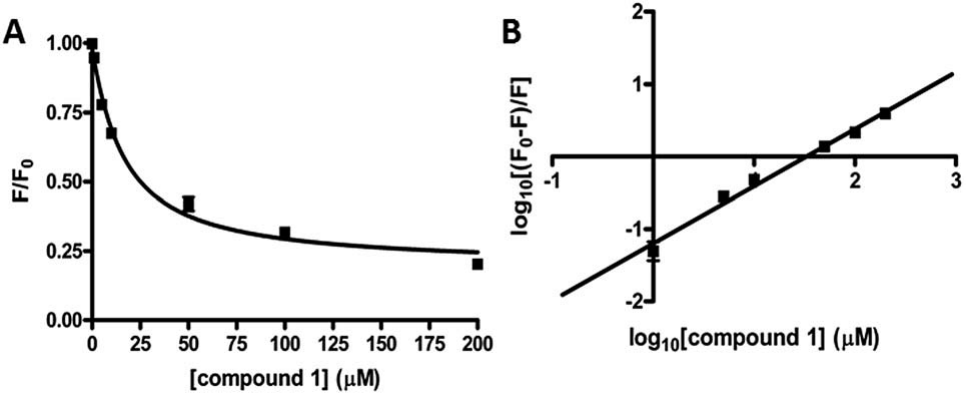
Binding of compound **1** to RocR. (A) Normalized RocR intrinsic fluorescence change with different concentrations of compound **1**, indicating a dissociation constant *K*_d_ of 14 ± 2 μM (eqn (1)†).^47^ (B) Stern–Volmer plot of the RocR intrinsic fluorescence. *K*_d_ was calculated as 16 ± 2 μM using eqn (2),^48^ see Methods section.†

We performed kinetic and thermodynamic experiments to determine binding and inhibition parameters of compound **1** against RocR. A Lineweaver–Burk plot of the inverse of initial reaction speed against the inverse of c-di-GMP concentration at fixed concentration of compound **1** gave an inhibition constant (*K*_i_) of 83 ± 7 μM (Fig. 6). According to the Lineweaver–Burk plot, compound **1** is a non-competitive inhibitor (Fig. 6B). An apparent dissociation constant, *K*apparentd, of 15 μM (average from two different methods,^47^,^48^ see Fig. 7) was obtained by measuring the change of RocR intrinsic fluorescence in the presence of different concentrations of compound **1**.

### Effects of compound **1** on bacterial motility

As stated earlier, there are about 21 EAL domain PDEs in *P. aeruginosa* and 3 HD-GYP domain PDEs.^26^ Many of these PDEs remain uncharacterized. RocR is one of the most well-studied and most active phosphodiesterases found in *P. aeruginosa*.^36^ RocR is essential for *P. aeruginosa* acute infection; infection with *rocR* mutant *P. aeruginosa* did not have fatal effects on mice.^9^ In another catheter-associated urinary tract infection (CAUTI) model, *P. aeruginosa* strain that overexpressed RocR was observed to have less CFU in the bladders and kidneys of infected mice than wild type strain.^49^ Because in *P. aeruginosa* c-di-GMP phosphodiesterase mutants were viable (but avirulent) it is likely that PDE inhibitors will not be used for growth inhibition purpose but rather could be used to attenuate bacterial virulence. Indeed, even at high concentrations (100 μM), compound **1** did not kill *P. aeruginosa* (Fig. S2†). In line with the minor effects of *rocR* mutant and overexpression strains on biofilm, treatment of *P. aeruginosa* with compound **1** did not increase biofilm formation (Fig. S3†).

The ability to move on surfaces (swarming) or in a viscous mucous (swimming) is critical for the invasive virulence of *P. aeruginosa*. Bacterial motility apparatus, such as flagella, are considered as virulence factors; in a mice burn wound model, non-motile *P. aeruginosa* were able to proliferate in the burn wound but were unable to cause bacteraemia or systemic invasion, and therefore the infection was localized to the skin wound.^50^ Small molecules that inhibit bacterial motilities are of interest due to the potential to use such molecules to reduce bacterial virulence.^51^,^52^ Chan and co-workers found that caffeine inhibited PAO1 swarming at 0.3 mg ml^–1^, probably *via* inhibition of quorum sensing.^53^ Fukui and co-workers showed that anteiso-C15:0, which is a branched-chain fatty acid completely abolished PAO1 swarming at 5 μg ml^–1^.^54^

**Fig. 8:**
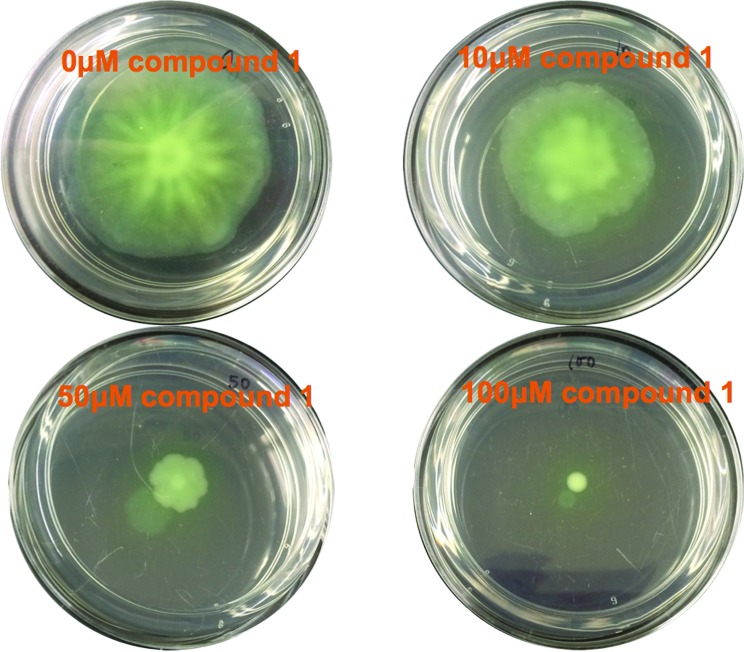
Swarming assay. Different concentrations of compound **1** (concentration indicated in the graph) were added to the swarming agar. 1 μl of PAO1 overnight culture was inoculated in the middle of the agar surface. Pictures were taken after 48 h incubation. The swarming mobility of PAO1 was reduced in the presence of compound **1** (10 μM and 100 μM).

*P. aeruginosa* swimming and swarming motilities are powered by a single polar flagellum,^55^ although others have proposed that swarmer cells might have two flagella.^56^,^57^ C-di-GMP mediates flagella biosynthetic gene repression *via* binding to a key transcription factor, FleQ.^58^*P. aeruginosa* encodes two stator complexes, MotAB (PA4954/4953) and MotCD (PA1460/1461).^59^ These stators are cytoplasmic membrane channels that generate flagella rotation torque by proton conduction.^60^ The numbers of MotAB and MotCD in a motor is dynamic and both MotAB and MotCD stators can provide energy for swimming motility. Disabling one stator did not completely abolish swimming.^59^ However MotAB and MotCD have opposite functions in swarmer cells.^60^ MotCD generates torque for swarming, but MotAB impedes it. *Via* an unknown mechanism, c-di-GMP downregulates the proportion of MotCD in a motor and slows down swarming.^61^ From the foregoing, an inhibitor of PDE could inhibit swarming and/or swimming. Compound **1** (at 10 μM) inhibited swarming, but not swimming. *P. aeruginosa* swarming was completely inhibited at 100 μM (see Fig. 8 and S4†). The swarming inhibition was dose-dependent (see Fig. 8), confirming that it is due to the direct action of compound **1**. In line with the observation that compound **1** did not inhibit swimming motility or biofilm formation, the global concentration of c-di-GMP did not change upon addition of compound **1** (see LC-MS/MS analysis, Fig. S5†).

**Fig. 9:**
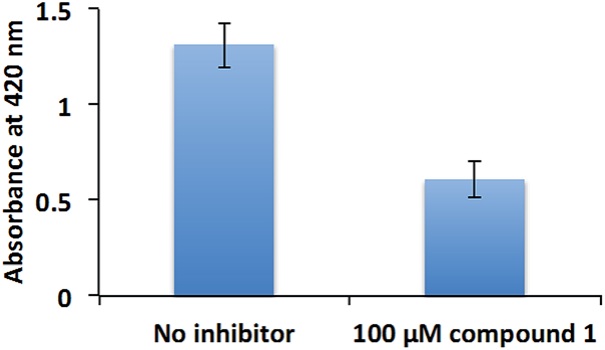
Rhamnolipid production. PAO1 was cultured with or without 100 μM compound **1**. Rhamnolipid was extracted with diethyl ether and evaporated to dryness. The extracted rhamnolipid was reacted with 0.19% (w/v) orcinol in 50% (v/v) concentrated H_2_SO_4_ at 80 °C for 30 min. The production of rhamnolipid was quantified by measuring absorbance at 421 nm.

The inhibition of swarming, but not swimming, by a compound is intriguing. At this stage we are unable to rule out if other non-RocR inhibition mechanisms also account for swarming inhibition because of the following: (i) not all PA PDEs have succumbed to expression and *in vitro* activity profiling (some c-di-GMP PDEs are membrane bound and non-trivial to express) and (ii) an experimental verification of localized changes of a metabolite in bacteria is non-trivial. Future experiments, which could utilize genetic and proteomic approaches (beyond the scope of this current study) should help identify other targets of compound **1** in bacteria. These opposite effects of compound **1** on swimming and swarming validate earlier findings by others, who have shown that although swimming and swarming utilize some common apparatus, there are some differences between these two.^62^ Swarming is a group behavior, whereas swimming is not.^63^ As the population of *P. aeruginosa* increases, so does the concentration of quorum sensing autoinducers, which promote the production of the surfactant rhamnolipid^56^ to aid swarming.^48^ Rhamnolipid is also a potent virulence factor, which is associated with ventilator-associated pneumonia,^64^ resistance to macrophage phagocytosis,^65^ respiratory epithelium invasion,^66^ host cell membrane disruption,^66^ among others. Therefore, we tested the rhamnolipid production of PAO1 in the presence or absence of compound **1** (Fig. 9). Compound **1** reduced the production of rhamnolipid, which is critical for PAO1 swarming motility.

## Conclusions

C-di-GMP has emerged as an interesting second messenger in bacteria and there is a high interest in finding small molecules that perturb c-di-GMP signaling in bacteria. The majority of reports on small molecule inhibitors of c-di-GMP signaling have focused on the inhibition of c-di-GMP synthesis,^67^–^69^ probably due to the central role that c-di-GMP plays in most Gram-negative bacterial biofilm maturation^1^ and resistance to stress.^70^ However c-di-GMP and its degradation product, pGpG, probably have complex roles in bacterial virulence factor production and a selective PDE inhibitor that could inhibit bacterial virulence but not affect biofilm formation has not been identified till date. Compound **1** did not inhibit other tested cyclic dinucleotide metabolic enzymes such as WspR D70E (c-di-GMP synthase from *P. aeruginosa*), DisA (c-di-AMP synthase, from *B. subtilis*) and GdpP (previously known as YybT, c-di-AMP phosphodiesterase from *B. subtilis*) (data not shown). Compound **1** also did not inhibit YahA (PDE from *E. coli*) or Snake venom phosphodiesterase (SVPD) or other c-di-GMP PDEs from *P. aeruginosa* (DipA, PvrR and PA4108) (see Fig. 5). Compound **1** therefore represents a first-in-class c-di-GMP PDE inhibitor that is cell-permeable and does not indiscriminately inhibit other key PDEs that regulate global c-di-GMP concentration. Future works will focus on improving the potency of compound **1** and also gaining structural insights of how compound **1** binds to RocR to inhibit the enzyme (*i.e.* X-ray crystal structure of compound **1**/RocR). Such studies could provide guiding principles to develop selective c-di-GMP PDE inhibitors that could be used to inhibit bacterial virulence but not increase biofilm formation.

## Supplementary Material

Supplementary information
