# Supplementary material for: Inhibition of P. aeruginosa c-di-GMP phosphodiesterase RocR and swarming motility by a benzoisothiazolinone derivative
Source: Chem Sci. 2016 Jun 16;7(9):6238–44. doi: 10.1039/c6sc02103d (PMC6024209; doi:10.1039/c6sc02103d)
Supplement: Supplementary file 1 [file SC-007-C6SC02103D-s001.pdf]

Electronic Supplementary Material (ESI) for Chemical Science.
This journal is © The Royal Society of Chemistry 2016

Supporting information

Inhibition of *P. aeruginosa* c-di-GMP phosphodiesterase RocR and swarming motility with a benzoisothiazolinone derivative.

Yue Zheng[a,b,c], Genichiro Tsuji[a,b], Clement Opoku-Temeng[a,b,c] and Herman O. Sintim[a,b]*
[a]Department of Chemistry, Purdue University, 560 Oval Drive, West Lafayette, IN 47907, USA.
[b]Center for Drug discovery, Purdue University, 720 Clinic Drive, West Lafayette, IN 47907 , USA.
[c]Department of Chemistry and Biochemistry, University of Maryland, College Park, MD 20742, USA.

Characterization spectrums

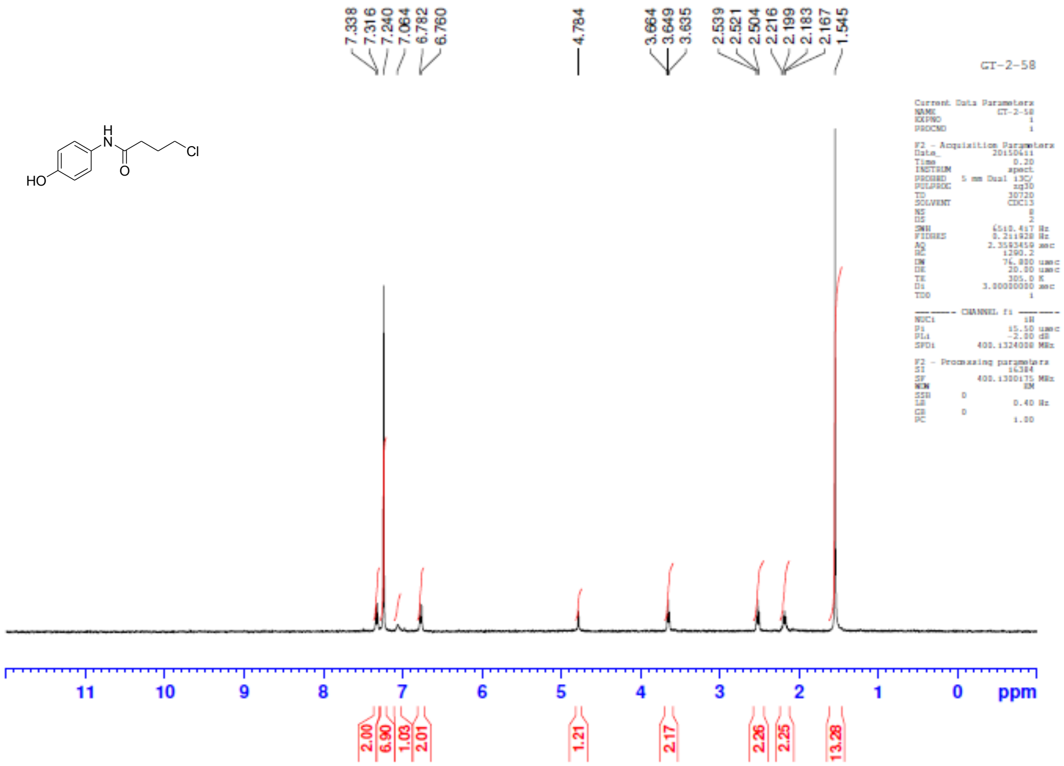

^{1}H NMR spectrum of **4**

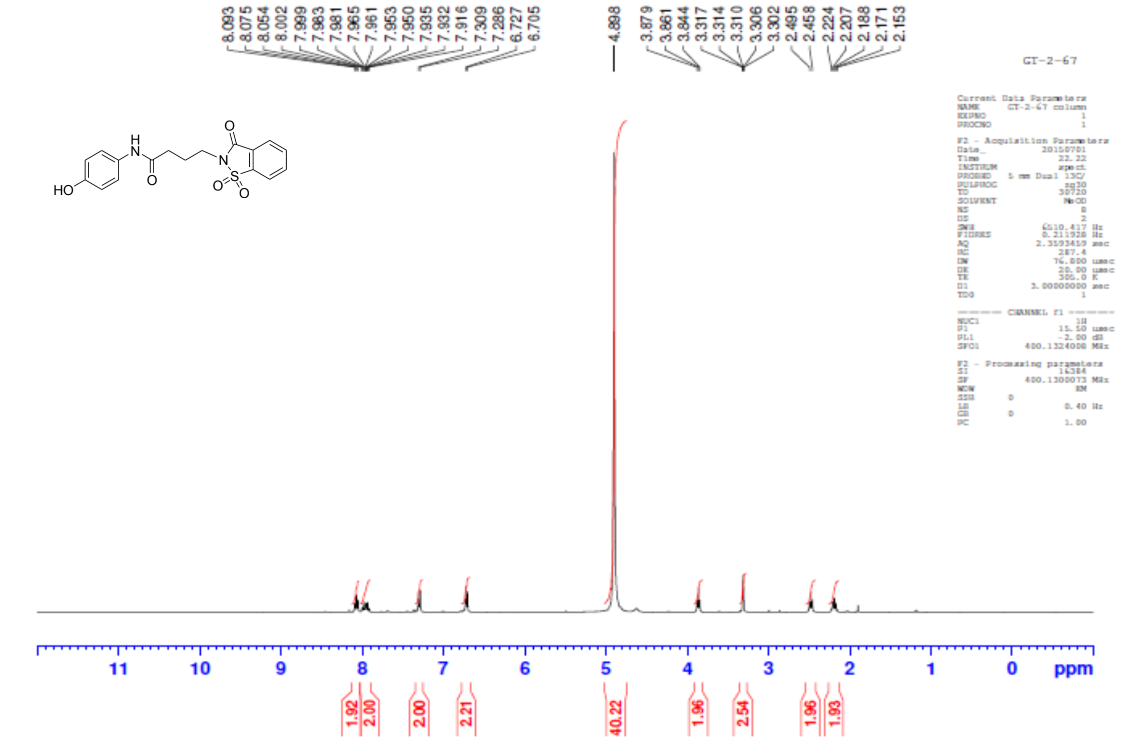

1H NMR spectrum of **2**

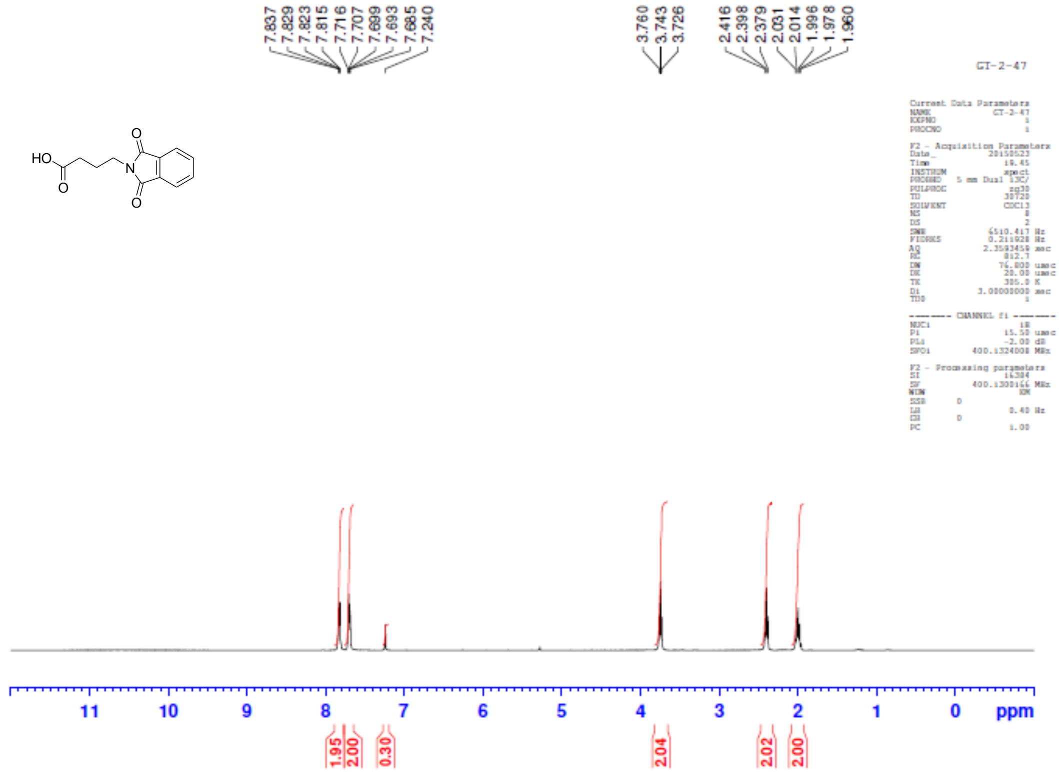

^{1}H NMR spectrum of **5**

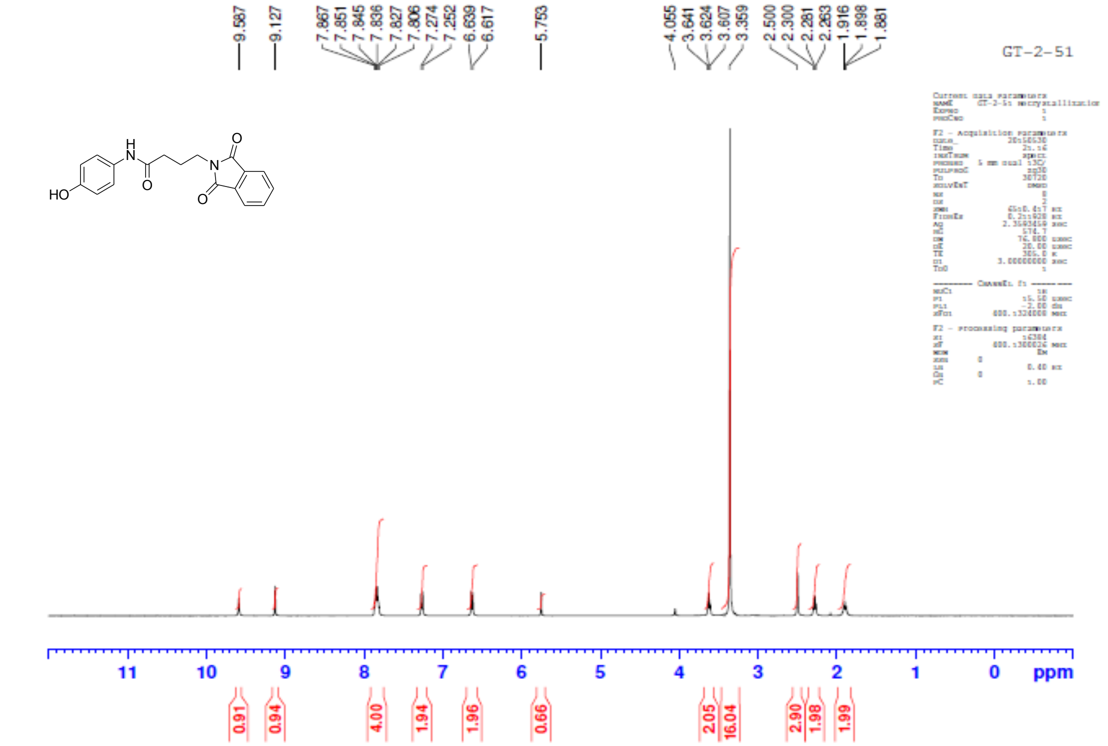

^{1}H NMR spectrum of **3**

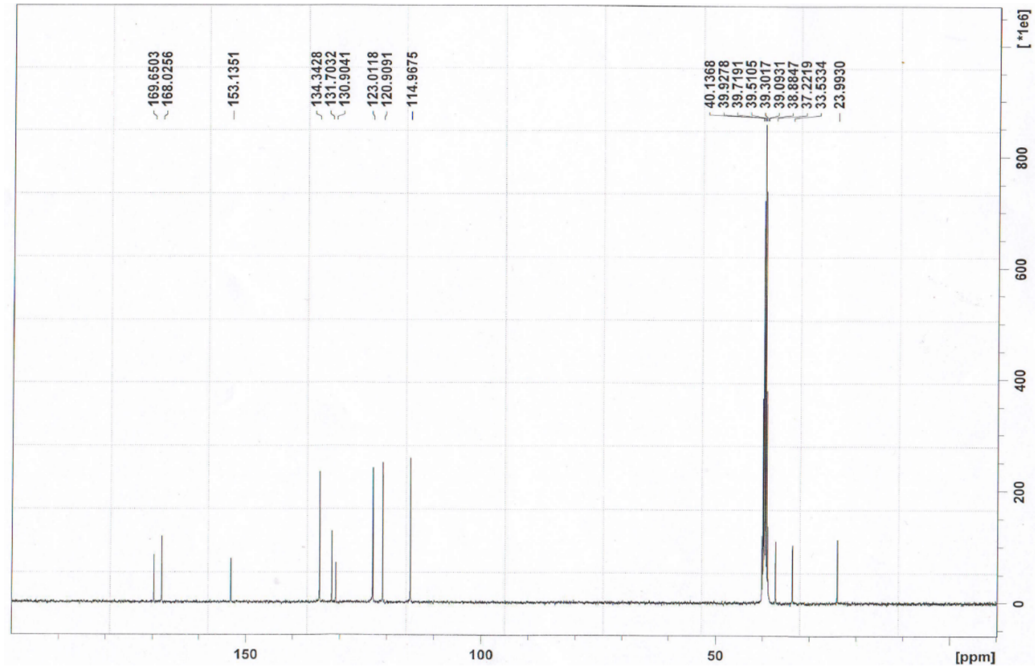

^{13}C NMR spectrum of **3**

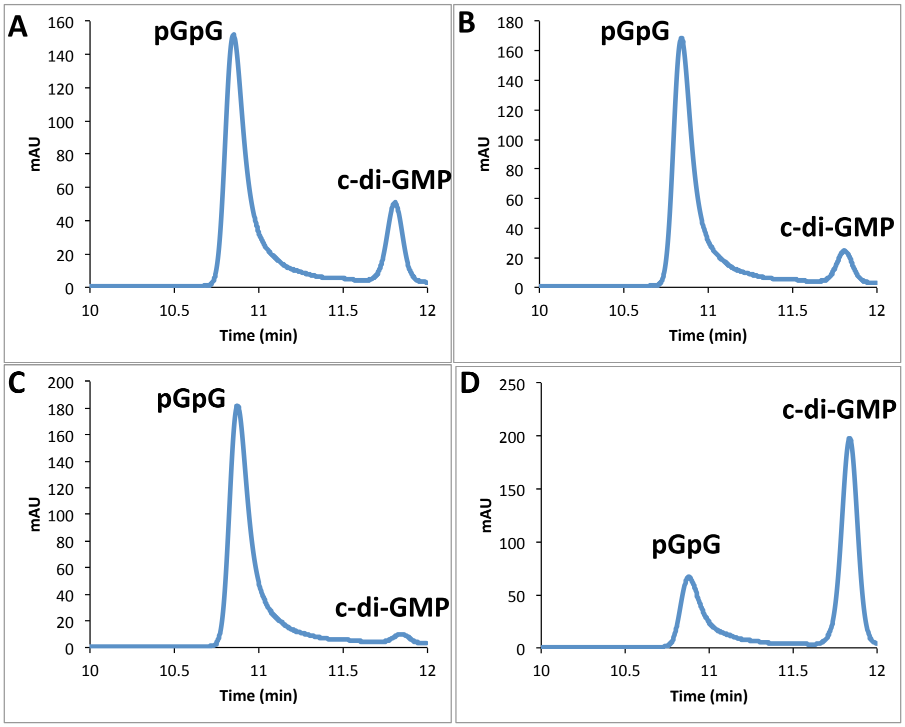

Fig. S1. Compound **1** inhibits RocR, but not YahA. (A)-(D) HPLC analysis of the cleavage of c-di-GMP by YahA and RocR in the presence of compound **1**. (A) 0.7 µM YahA and 50 µM c-di-GMP in the absence of compound **1**. (B) 0.7 µM YahA and 50 µM c-di-GMP in the presence of 100 µM compound **1**. (C) 0.6 µM RocR and 50 µM c-di-GMP in the absence of compound **1**. (D) 0.6 µM RocR and 50 µM c-di-GMP in the presence of 100 µM compound **1**. The reactions were conducted at 37 °C for 30 min.

Table S1. Selected PDEs from *P. aeruginosa* and the phenotypes that they regulate

Name	Intracellular c-di-GMP level	*In vitro* activity	Phenotype
NbdA[1]	Δ*nbdA* did not alter c-di-GMP levels but overexpression of *nbdA* reduced c-di-GMP level.	Yes	Δ*nbdA* is deficient in biofilm dispersal
MucR[1]	Δ*mucR* increased c-di-GMP level in the biofilm cells, but decreased c-di-GMP level the planktonic cells	Yes	Δ*mucR* is deficient in biofilm dispersal
BifA[2]	Δ*bifA* increased c-di-GMP level	Yes	Δ*bifA* reduced swarming motility and increased biofilm formation
RocR[3, 4]	N/A	Yes	*rocR* mutant reduced virulence, *rocR* mutant and overexpression strains did not affect biofilm
DipA[5]	Δ*dipA* increased c-di-GMP level	Yes	Δ*dipA* is deficient in biofilm dispersal, Δ*dipA* reduced swarming motility

PvrR[6, 7]	*pvrR* overexpression reduced c-di-GMP level	Yes	*pvrR* overexpression reduced biofilm formation, *pvrR* mutant reduced virulence
PA4108[8]	PA4108 mutant increased c-di-GMP level	Yes	PA4108 mutant reduced swarming motility and pyocyanin production
PA4781[8]	PA4781 mutant increased c-di-GMP level	Yes	PA4781 mutant reduced swarming motility and increased pyoverdin production

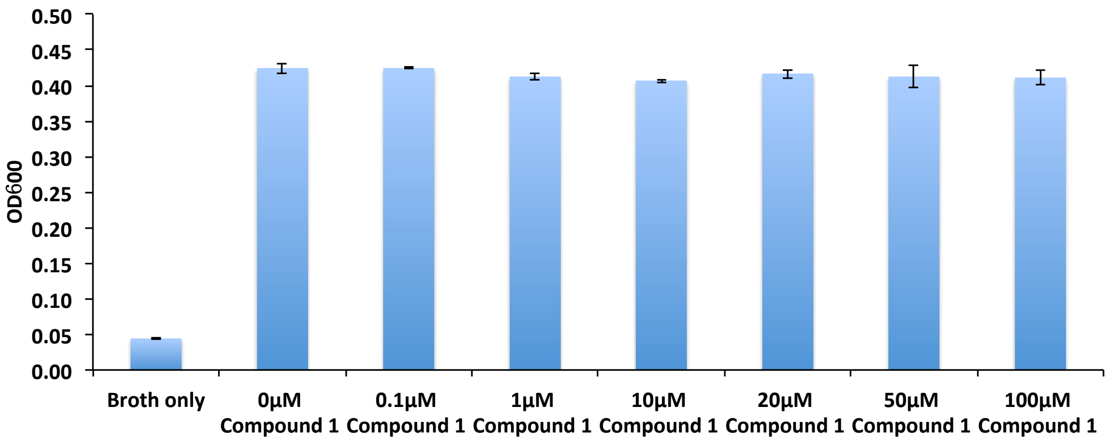

Fig. S2. Viability tests of compound **1** on PAO1. PAO1 was treated with different concentrations of compound **1**. Compound **1** did not kill PAO1 cells.

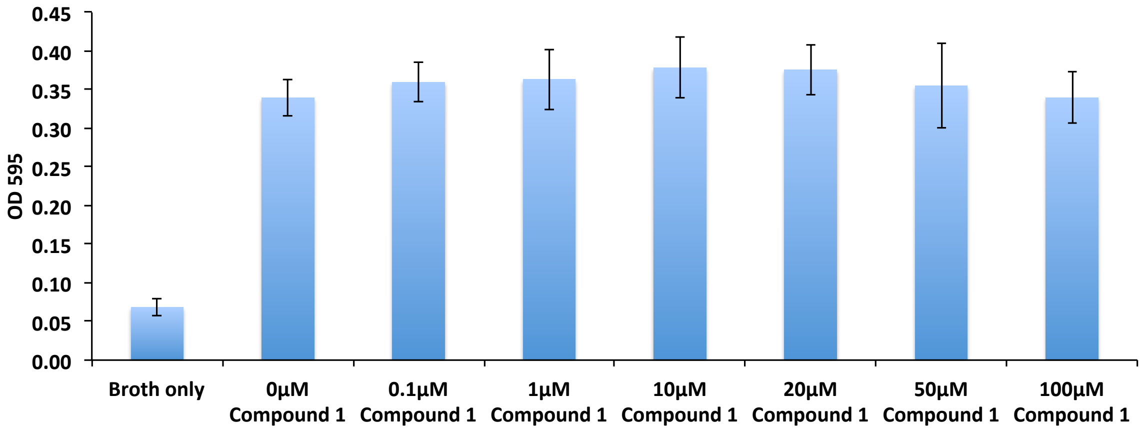

Fig. S3. Compound **1** did not affect PAO1 biofilm. PAO1 was treated with different concentrations of compound **1**, but biofilm formation was not inhibited by compound **1**.

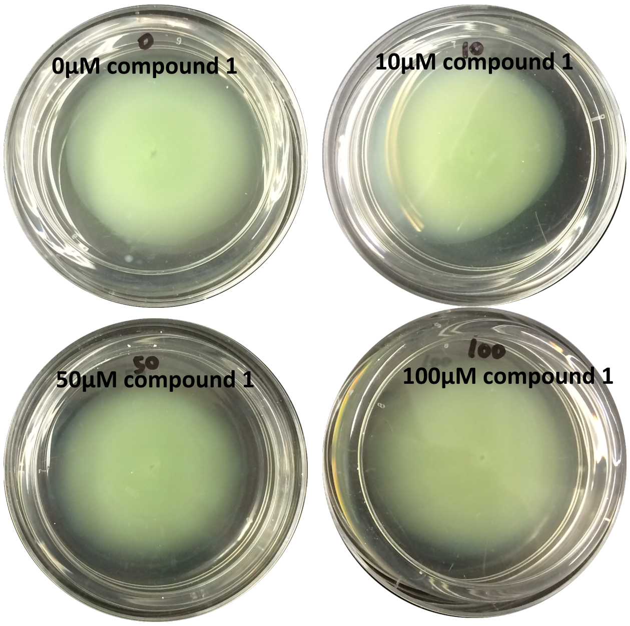

Fig. S4. Swimming assay. Different concentrations of compound **1** (concentration indicated in the graph) were added to the swimming agar. PAO1 overnight culture was inoculated into the agar by a needle. Pictures were taken after 24 h incubation. Compound **1** did not inhibit PAO1 swimming motility.

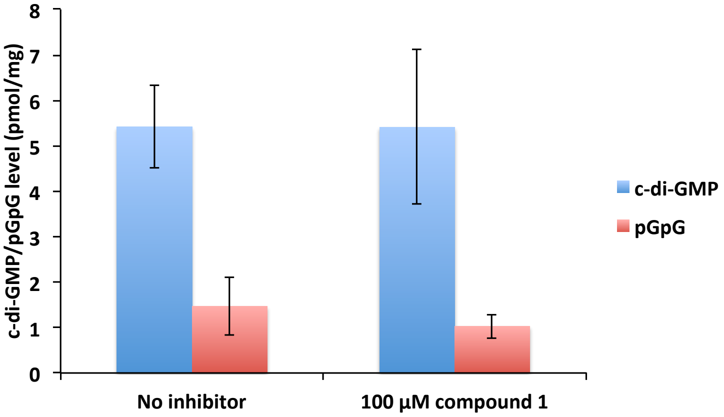

Fig. S5. PAO1 intracellular concentrations of c-di-GMP and pGpG in the presence or absence of compound **1**. C-di-GMP and pGpG concentration were quantified by LC-MS/MS. For the details of extraction and quantification, see Methods section.

Methods

Protein purification. Snake venom phosphodiesterase (SVPD) from *Crotalus atrox* was purchased from Sigma-Aldrich. RocR, WspR, DisA, YahA and YybT purification were performed as described previously.[9] DipA, PA4108 and PvrR plasmids were transformed into BL21 (DE3) cells. When OD_{600} got to 0.6, protein expression was induced by 1 mM IPTG. After expression at 16 °C overnight, cells were harvested by centrifugation

(Sorvall LYNX 6000 Superspeed Centrifuge) at 5000 rpm for 30 min. Cells were resuspended in lysis buffer and lysed by sonication. Cell lysates were centrifuged at 22,000 rpm for 25 min. Proteins were purified from supernatant by Nickel column (GE Healthcare HiTrap column). Lysis buffer for DipA contains 10 mM Tris-HCl, pH 8.0, 1 mM EDTA and 0.1% Tween 20. Lysis buffer for PA4108 contains 50 mM Tris-HCl, pH 8.0 and 50 mM NaCl. Lysis buffer for PvrR contains 10 mM Tris-HCl, pH 8.0 and 100 mM NaCl.

Radiolabeling experiments. ^{32}P-c-di-GMP was synthesized by incubating ^{32}P-GTP (333 nM), GTP (20 μM) and WspR D70E (5 μM) in a buffer containing 10 mM Tris-HCl, pH 8.0, 100 mM NaCl and 5 mM $MgCl_2$ at 37 °C overnight. For inhibition tests, c-di-GMP (50 μM) and ^{32}P-c-di-GMP (16 nM) were cleaved by RocR or YahA in the presence or absence of 100 μM potential inhibitors at 37 °C. The RocR reaction buffer, pH 8.0, contained 100 mM Tris-HCl, 20 mM KCl and 25 mM $MgCl_2$. YahA reaction buffer pH, 9.35, contained 50 mM Tris-HCl, 5 mM $MgCl_2$ and 50 mM NaCl. At different time points, 0.5 μL of reaction mixture was applied on a cellulose TLC plate (EMD Millipore). The TLC plates were developed in a buffer consisting of 1:1.5 (v/v) mixture of saturated $(NH_4)_2SO_4$: 1.5 M KH_2PO_4. To measure the kinetics of inhibition, radiolabeling experiments were conducted to monitor the reaction initial velocities with different concentrations of c-di-GMP and a fixed concentration of RocR (600 nM). 50 μM of compound **1** was used to determine K_i.

Bis-pNPP cleavage assays. 0.77 μM DipA or 4.7 μM PA4108 or 5 μM PvrR or 0.001 unit/mL SVPD reacted with 1 mM bis-pNPP at 37 °C for 1 h in the presence or absence of 100 μM compound **1**. OD_{420} was monitored by BioTek Cytation 5 Cell Imaging Multi-Mode Reader.

Dissociation constant K_d measurement. 5 μM RocR and different concentrations of c-di-GMP were incubated at 4 °C for 1 h. Protein intrinsic fluorescence measurement (Excitation: 280 nm and Emission: 300 ~ 430 nm) was carried out on a Varian Cary Eclipse fluorescence spectrophotometer.

The dissociation constant was calculated by the following equations:

$$\mathrm{F} = \mathrm{F_0} + \Delta \mathrm{F}\frac{\left(K_d^{app} + \mathrm{P_t} + \mathrm{L_t}\right) - \sqrt{(K_d^{app} + \mathrm{P_t} + \mathrm{L_t})^2 - 4\mathrm{P_t}\mathrm{L_t}}}{2\mathrm{P_t}} \qquad (1)^{10}$$

$$\log\left(\frac{\mathrm{F_0}\text{-}\mathrm{F}}{\mathrm{F}}\right) = \log K_a + \mathrm{n}\log[\mathrm{L_t}] \qquad (2)^{11}$$

Where F is the intrinsic fluorescence intensity at 340 nm. F_0 is the fluorescence intensity at 340 nm in the absence of compound **1**. ΔF is the fluorescence intensity change upon compound **1** binding. K_a is the association constant and K_d^{app} is the apparent dissociation constant of binding of compound **1** to RocR. P_t is the total protein concentration and L_t is the total compound **1** concentration.

Intracellular c-di-GMP and pGpG concentration quantification. PAO1 overnight culture was diluted 1:100 into fresh TSB medium. 5 mL of the diluted PAO1 cultures with or without 100 μM compound **1** were shaken at 37 °C for 24 h. Cells were harvested by centrifugation (Sorvall LYNX 6000 Superspeed Centrifuge) at 5000 rpm for 30 min. Cells were resuspended in 10 mM Tris-HCl, pH 8.0 and 100 mM NaCl and lysed by sonication. After centrifugation at 12,000 rpm for 10 min, the supernatant was evaporated to dryness in SpeedVac. The pellets were extracted with a mixed solvent containing methanol: CH_3CN: water at 2:2:1 for 3 times. Protein concentration was determined using the pellet fraction. All of the extracted supernatants were combined, filtered through 10K cut-off filter and evaporated to a final volume of 50 μL. cXMP was added to the supernatant (to make a final concentration of 100 nM) as internal standard and c-di-GMP and pGpG concentrations were quantified with Agilent 6460 Triple Quadrupole LC-MS/MS system using a Waters Atlantis T3 Column. Buffer A contains 0.1% formic acid in water and buffer B contains 0.1% in acetonitrile. The gradient is 0 → 16 min: 0% buffer B → 80% buffer B.

Viability test. *P. aeruginosa* PAO1 overnight culture was diluted to an OD_{600} of 0.02 in LB medium. 200 μL of bacterial culture was added to a 96 well microplate with 0, 0.1 μM, 1 μM, 10 μM, 20 μM, 50 μM or 100 μM compound **1**. After 24 h incubation at 37 °C, OD_{600} was measured by Molecular devices M5e microplate reader. Viability tests were done in triplicates.

Swimming and swarming assays. For swimming assay, 3 g agar in 800 mL deionized water was autoclaved and then mixed with sterile 200 mL of 5×M8 medium, 25 mL of 20% casamino acids, 10 mL of 20% glucose and 1 mL of 1 M $MgSO_4$.[12] 7.5 mL of mixed swimming agars with different concentrations of compound **1** were poured into 60 mm plates. The plates were dried for 30 min. PAO1 overnight culture was inoculated into the agar plates via an inoculating needle. Swimming plates were incubated at 37 °C for 24 h. For swarming assay, the components of the swarming agar were the same as swimming agar except that 5 g agar was added.[13] After drying for 30 min, 1 μL of PAO1 overnight culture was added onto the agar surface. Swarming plates were incubated at 37 °C for 24 h and then at room temperature for 24 h. Swimming and swarming experiments were done in duplicates.

Rhamnolipid determination. Rhamnolipid was determined according to the methods published by Blackwell.[14] *P. aeruginosa* PAO1 was grown in 15 mL LB medium overnight. Minimal Medium containing 49.3 mM Na_2HPO_4, 50 mM KH_2PO_4, 4.8 mM $MgSO_4$, 7.6 mM $(NH_4)_2SO_4$, 0.6 mM $CaCl_2$, 25 μM $FeSO_4$, 0.162 μM $(NH_4)_6Mo_7O_{24}$, 38 μM $ZnSO_4$, 14 μM $MnCl_2$, 1.6 μM $CuSO_4$, 0.86 μM $CoCl_2$, 1.9 μM boric acid, 5.5 μM $NiCl_2$, 6.72 μM EDTA, 0.6% glycerol in water was then used for the next step. PAO1 overnight culture was diluted 1:100 into the fresh Minimal Medium. DMSO or 100 μM compound **1** was added to the culture. After 20 h shaking at 37 °C, cell culture supernatant was collected by centrifugation at 5000 rpm for 20 min using Sorvall LYNX 6000 Superspeed Centrifuge. Rhamonolipid was extracted with diethyl ether, evaporated to dryness and re-dissolved in 200 μL water. 50 μL of rhamnolipid solution was mixed with 450 μL of 0.19% (w/v) orcinol in 50% (v/v) concentrated H_2SO_4. After incubation at 80 °C for 30 min, the solutions were allowed to cool at RT for 15 min and the absorbance

at 421 nm was measured by Jasco V-730 UV-Visible/NIR Spectrophotometer. Data was normalized to OD_{600} of PAO1 culture.

Biofilm assay. *P. aeruginosa* PAO1 overnight culture was diluted to an OD_{600} of 0.02 in 10 % TSB. 200 μL of bacterial culture was added to a MBEC plate (Innovotech) with 0, 0.1 μM, 1 μM, 10 μM, 20 μM, 50 μM or 100 μM compound **1**. After 4 hour incubation at 37 °C, pegs were stained with 0.1% crystal violet and washed three times with distilled water. Then air-dried pegs were dipped into ethanol until crystal violet was completely dissolved. OD_{595} was measured by Molecular devices M5e microplate reader. Biofilms assays were repeated eight times.

General Chemical Syntheses and Methods for characterization. All chemicals were used as received. Reactions were monitored by thin layer chromatography (TLC) using F-254 silica gel aluminum backed plates (Dynamic Adsorbents, Inc.). All NMR spectra were recorded on a Bruker AV-400 or Bruker DRX-400. Measurements were performed at room temperature in deuterated solvent using the residual solvent hydrogen resonances as standards ($CDCl_3$: δ = 7.24 ppm; DMSO-d_6 : δ = 2.50 ppm; CD_3OD : δ = 3.31 ppm). Chemical shifts are expressed in parts per million (ppm, δ units). Coupling constants (J) are in units of hertz (Hz). Splitting patterns describe apparent multiplicities and are designated s (singlet), d (doublet), t (triplet), dd (double doublet), m (multiplet), and br (broad). ^{13}C-NMR chemical shifts are reported as ppm relative to residual solvent peak. High-resolution mass spectra (HRMS) for synthesized compounds were recorded with JEOL AccuTOF-CS (ESI positive, needle voltage 1800~2400 eV). Compound **1** was purchased from Chembridge Corporation. Compound **2** and **3** were prepared by following the synthesis scheme in Figure 3.

N-(4-Hydroxyphenyl)-4-chlorobutyrylamide (**4**)[15]

4-aminophenol (2.18 g, 20.0 mmol) was suspended in a solution of acetic acid (20 mL)/saturated solution of sodium acetate (20 mL). To this solution was cooled to 0 °C, and added dropwise 4-chlorobutyryl chloride (2.13 mL, 24.0 mmol) under stirring. After completion of addition, the reaction mixture was stirred for 2 h at that temperature. The resulting precipitate was collected by filtration, and washed with cold water (50 mL), and dried under vacuum. The obtained solid was washed with a solution of hexanes/diethyl ether (5:1, v/v), and dried under reduced pressure to give a white floc solid (3.21 g, 75%)

^{1}H NMR(400 MHz, $CDCl_3$) δ : 7.32 (2H, d J = 8.8 Hz), 3.74 (1H, br. s), 6.77 (2H, d, J = 8.8 Hz), 3.74 (1H, s), 3.65 (2H, t, J = 6.4 Hz), 2.52 (2H, t, J = 6.4 Hz), 2.20 – 2.17 (2H, m)

N-(4-Hydroxyphenyl)-4-[2,3-dihydro-3-oxo-1,2-benzisothiazol-2-yl-1,1-dioxide]-butyrylamide (**2**)[15]

To a solution of *o*-sulfobenzimide (183 mg, 1.00 mmol) in DMF (2 mL) was added NaH (60% oil dispersion)(44 mg, 1.10 mmol) in one portion at 0 °C under stirring. After stirring at that temperature for 30 min at that temperature, the reaction mixture was added to **4** (143 mg, 0.67 mmol), sodium iodide (30 mg, 0.20 mmol). The reaction mixture was stirred at 80 °C for 2 h. After cooling to rt, the reaction mixture was diluted with EtOAc, and washed with 1 M HCl, brine. The organic layer was dried over Na_2SO_4, and

concentrated under vacuum to afford a brown solid. This product was purified by silica gel column chromatography (n-Hexane/EtOAc = 2 : 1 to 1 : 1) to afford a light brownish solid (85 mg, 24%).

^{1}H NMR(400 MHz, CD_3OD) δ : 8.08 (2H, dd), 7.98 – 7.92 (2H, m), 7.30 (2H, d, J = 9.0 Hz), 6.71 (2H, d, J = 9.0 Hz), 3.85 (2H, t, J = 6.8 Hz), 2.47 (2H, t, J = 6.8 Hz), 2.22 – 2.15 (2H, m)

HRMS (m/z): calcd. for $C_{17}H_{17}N_2O_5S$: 361.0858 $[M+H]^+$, found 361.0836 $[M+H]^+$

4-(1,3-Dioxoisoindolin-2-yl)butanoic acid (**5**)[16]

A mixture of phthalic anhydride (1.48 g, 10.0 mmol), 4-aminobutyric acid (1.08 mg, 10.5 mmol), acetic acid (0.57 mL, 10.0 mmol) in toluene (10 mL) was stirred at 110 °C for 16 h. After cooling to rt, the reaction mixture was diluted with EtOAc, and washed with 1 M HCl, brine. The organic layer was dried over Na_2SO_4, and concentrated under vacuum to afford a white solid. This product was further purified by recrystallization from n-Hexane/Et_2O to give a colorless crystalline powder (1.235 g, 53%).

^{1}H NMR(400 MHz, $CDCl_3$) δ : 4.67 (1H, brs), 3.20 – 3.17 (2H, m), 2.74 (2H, t, J = 6.4 Hz), 1.62 – 1.55 (2H, m), 1.42 (9H, s)

4-(1,3-dioxoisoindolin-2-yl)-*N*-(4-hydroxyphenyl)butanamide (**3**)

To a suspension of **5** (117 mg, 0.50 mmol), 1-hydroxy-7-azabenzotriazole (HOAt) (68 mg, 0.50 mmol) in CH_2Cl_2 (5 mL) was added 1-(3-dimethylaminopropyl)-3-ethylcarbodiimide hydrochloride (EDC-HCl) (145 mg,0.75 mmol), and stirred at rt for 30 min. To the reaction mixture was added 4-aminophenol (58 mg, 0.53 mmol), and stirred at rt for 4 h. The reaction mixture was diluted with EtOAc (50 mL), and washed with 0.5M HCl aq., brine. The organic layer was dried over Na_2SO_4, and concentrated under vacuum to afford a white solid. This product was purified by recrystallization from EtOAc/EtOH to give a white floc solid (55 mg, 34%).

^{1}H NMR(400 MHz, DMSO-d_6) δ : 9.59 (1H, br. s), 9.13 (1H, s), 7.85 – 7.83 (4H, m), 7.26 (2H, d, J = 8.8 Hz), 6.62 (2H, d, J = 8.8 Hz), 5.75 (1H, s), 3.62 (2H, t, J = 6.8 Hz), 2.28 (2H, t, J = 7.4 Hz), 1.92 – 1.88 (2H, m)

^{13}C NMR(101 MHz, DMSO-d_6) δ : 169.7, 168.0, 153.1, 134.3, 131.7, 130.9, 123.0, 120.9, 115.0, 37.2, 33.5, 24.0

HRMS (m/z): calcd. for $C_{18}H_{17}N_2O_4$: 325.1188 $[M+H]^+$, found : 325.1156 $[M+H]^+$

Reference:

1. Y. Li, S. Heine, M. Entian, K. Sauer and N. Frankenberg-Dinkel, *J. Bacteriol.*, 2013, **195**, 3531-3542.
2. S. L. Kuchma, K. M. Brothers, J. H. Merritt, N. T. Liberati, F. M. Ausubel and G. A. O'Toole, *J. Bacteriol.*, 2007, **189**, 8165-8178.
3. F. Rao, Y. Yang, Y. Qi and Z. X. Liang, *J. Bacteriol.*, 2008, **190**, 3622-3631.
4. H. Kulasakara, V. Lee, A. Brencic, N. Liberati, J. Urbach, S. Miyata, D. G. Lee, A. N. Neely, M. Hyodo, Y. Hayakawa, F. M. Ausubel and S. Lory, *Proc. Natl. Acad. Sci. U. S. A.*, 2006, **103**, 2839-2844.
5. A. B. Roy, O. E. Petrova and K. Sauer, *J. Bacteriol.*, 2012, **194**, 2904-2915.
6. H. Mikkelsen, G. Ball, C. Giraud and A. Filloux, *PLoS One*, 2009, **4**, e6018.

7. A. Meissner, V. Wild, R. Simm, M. Rohde, C. Erck, F. Bredenbruch, M. Morr, U. Römling and S. Häussler, *Environ. Microbiol.*, 2007, **9**, 2475-2485.
8. R. P. Ryan, J. Lucey, K. O'Donovan, Y. McCarthy, L. Yang, T. Tolker-Nielsen and J. M. Dow, *Environ. Microbiol.*, 2009, **11**, 1126-1136.
9. Y. Zheng, J. Zhou, D. A. Sayre and H. O. Sintim, *Chem. Commun. (Camb)*, 2014, **50**, 11234-11237.
10. C. Bodenreider, D. Beer, T. H. Keller, S. Sonntag, D. Wen, L. Yap, Y. H. Yau, S. G. Shochat, D. Huang, T. Zhou, A. Caflisch, X. C. Su, K. Ozawa, G. Otting, S. G. Vasudevan, J. Lescar and S. P. Lim, *Anal. Biochem.*, 2009, **395**, 195-204.
11. K. M. Naik, D. B. Kolli and S. T. Nandibewoor, *Springerplus*, 2014, **3**, 360.
12. D. G. Ha, S. L. Kuchma and G. A. O'Toole, *Methods Mol. Biol.*, 2014, **1149**, 59-65.
13. D. G. Ha, S. L. Kuchma and G. A. O'Toole, *Methods Mol. Biol.*, 2014, **1149**, 67-72.
14. M. A. Welsh, N. R. Eibergen, J. D. Moore and H. E. Blackwell, *J. Am. Chem. Soc.*, 2015, **137**, 1510-1519.
15. A. L. Vaccarino, D. Paul, P. K. Mukherjee, E. B. Rodríguez de Turco, V. L. Marcheselli, L. Xu, M. L. Trudell, J. M. Minguez, M. P. Matía, C. Sunkel, J. Alvarez-Builla and N. G. Bazan, *Bioorg. Med. Chem.*, 2007, **15**, 2206-2215.
16. P. Ahuja, A. Husain and N. Siddiqui, *Med. Chem. Res.*, 2014, **23**, 4085-4098.
